# Construction and evaluation of a risk model for adverse outcomes of necrotizing enterocolitis based on LASSO-Cox regression

**DOI:** 10.3389/fped.2024.1366913

**Published:** 2024-10-07

**Authors:** HaiJin Zhang, RongWei Yang, Yuan Yao

**Affiliations:** ^1^ Department of Radiology, Jiaxing Maternity and Child Health Care Hospital, Jiaxing, Zhejiang, China; ^2^ Department of Pediatrics, Jiaxing Maternity and Child Health Care Hospital, Jiaxing, Zhejiang, China

**Keywords:** necrotizing enterocolitis, risk factors, prediction, abdominal x-ray, Cox proportional hazards regression, nomogram

## Abstract

**Objective:**

This study aimed to develop a nomogram to predict adverse outcomes in neonates with necrotizing enterocolitis (NEC).

**Methods:**

In this retrospective study on neonates with NEC, data on perinatal characteristics, clinical features, laboratory findings, and x-ray examinations were collected for the included patients. A risk model and its nomogram were developed using the least absolute shrinkage and selection operator (LASSO) Cox regression analyses.

**Results:**

A total of 182 cases of NEC were included and divided into a training set (148 cases) and a temporal validation set (34 cases). Eight features, including weight [*p* = 0.471, HR = 0.99 (95% CI: 0.98–1.00)], history of congenital heart disease [*p* < 0.001, HR = 3.13 (95% CI:1.75–5.61)], blood transfusion before onset [*p* = 0.757, HR = 0.85 (95%CI:0.29–2.45)], antibiotic exposure before onset [*p* = 0.003, HR = 5.52 (95% CI:1.81–16.83)], C-reactive protein (CRP) at onset [*p* = 0.757, HR = 1.01 (95%CI:1.00–1.02)], plasma sodium at onset [*p* < 0.001, HR = 4.73 (95%CI:2.61–8.59)], dynamic abdominal x-ray score change [*p* = 0.001, HR = 4.90 (95%CI:2.69–8.93)], and antibiotic treatment regimen [*p* = 0.250, HR = 1.83 (0.65–5.15)], were ultimately selected for model building. The C-index for the predictive model was 0.850 (95% CI: 0.804–0.897) for the training set and 0.7880.788 (95% CI: 0.656–0.921) for the validation set. The area under the ROC curve (AUC) at 8-, 10-, and 12-days were 0.889 (95% CI: 0.822–0.956), 0.891 (95% CI: 0.829–0.953), and 0.893 (95% CI:0.832–0.954) in the training group, and 0.812 (95% CI: 0.633–0.991), 0.846 (95% CI: 0.695–0.998), and 0.798 (95%CI: 0.623–0.973) in the validation group, respectively. Calibration curves showed good concordance between the predicted and observed outcomes, and DCA demonstrated adequate clinical benefit.

**Conclusions:**

The LASSO-Cox model effectively identifies NEC neonates at high risk of adverse outcomes across all time points. Notably, at earlier time points (such as the 8-day mark), the model also demonstrates strong predictive performance, facilitating the early prediction of adverse outcomes in infants with NEC. This early prediction can contribute to timely clinical decision-making and ultimately improve patient prognosis.

## 1 Introduction

In recent years, the survival rate of premature infants in the neonatal intensive care unit (NICU) has been steadily increasing due to the introduction of surfactant therapy and improvements in respiratory distress syndrome care. However, necrotizing enterocolitis (NEC) has emerged as a significant and devastating disease in this population, characterized by substantial morbidity, mortality, and financial burden (1). Clinical manifestations of NEC primarily include vomiting, diarrhea, and bloody stools. In severe cases, it can result in gastrointestinal perforation, multiple organ dysfunction, and life-threatening conditions for neonates. Approximately 7% of neonates with very low birth weight (VLBW) admitted to the NICU are at risk of developing NEC (2). Surgical intervention becomes necessary in 30%–40% of NEC cases, with an overall mortality rate of around 25% among neonates undergoing surgery (3). Furthermore, a notable proportion of patients may experience long-term complications, such as intestinal stenosis, short bowel syndrome, and neurodevelopmental abnormalities (4). These complications are closely associated with factors such as intestinal ischemia-hypoxia, infection, intestinal dysbiosis, improper feeding practices, and inappropriate timing of surgical intervention.

According to the severity of the disease and treatment needs, NEC can be divided into the medical treatment group and the surgical intervention group. In the traditional surgical intervention group for NEC, intestinal perforation is usually considered an absolute indication for surgery. However, performing surgical intervention after the occurrence of abdominal distension in newborns has missed the optimal treatment window, increasing the risk of postoperative death and complications. Previous studies (5–7) have shown that proactive surgical intervention before the occurrence of abdominal distension can reduce adverse outcomes and mortality in NEC, effectively control systemic metabolic disorders, and reduce postoperative complications such as intestinal stenosis. Therefore, early detection of NEC patients who require surgical treatment and timely surgical intervention are crucial.

The pathophysiological factors underlying the development of NEC are highly complex, and researchers have made efforts to identify various factors associated with poor prognosis. However, accurately predicting the prognosis of neonatal NEC and determining the optimal timing for surgery remain challenging (8–10). Several risk factors, including clinical features, routine laboratory and radiological findings (11–13), as well as novel microbiological and metabolomic biomarkers (14, 15), have been investigated. Despite these efforts, the predictive value of single risk factors remains limited (16), and even novel biomarkers, while potentially prognostic, require further development for clinical application (17). To address these limitations, some researchers have attempted to construct predictive scoring systems and machine learning models incorporating multiple factors (18–20). These studies indicate that integrating clinical presentation, laboratory results, and imaging findings, or using predictive models constructed from these features, can support NEC prognosis assessment to some extent and provide valuable guidance for clinical decision-making. Nonetheless, it is important to recognize that existing methods and models each have their own strengths and limitations. Consequently, there are currently no mature predictive models in clinical use for the prognosis assessment of NEC patients, highlighting the need for further research.

This study integrated a relatively comprehensive range of factors including perinatal, clinical, laboratory, and abdominal x-ray findings to construct a Cox proportional hazards regression model and a nomogram for assessing the risk and predicting adverse outcomes in neonatal necrotizing enterocolitis (NEC). The predictive performance of the model was evaluated. The aim is to develop a novel predictive model that provides decision support for clinicians in predicting the optimal timing for surgical intervention, ultimately improving the prognosis of NEC patients.

## 2 Materials and methods

### 2.1 Study subjects

This retrospective study included neonates diagnosed with NEC admitted to NICU at Jiaxing Maternal and Child Health Care Hospital, affiliated with Jiaxing College from January 1, 2017, to December 31, 2022. Inclusion criteria were as follows: (1) neonates with NEC diagnosed according to the Vermont Oxford Network Definition (VOND) criteria (21). (2) patients with complete clinical and surgical data, and (3) patients who underwent x-ray bedside examinations at 6-h intervals. The exclusion criteria were as follows: (1) neonates with severe complications who died within 48 h of admission, (2) neonates with congenital gastrointestinal malformations, (3) neonates with isolated spontaneous intestinal perforation (SIP), and (4) neonates with pneumoperitoneum detected on the initial x-ray examination. The data used for model development set included records from January 1, 2017, to December 31, 2021. Retrospectively collected data from January 1, 2022, to December 31, 2022, meeting the same inclusion criteria, were utilized for temporal validation of the developed model.

This study was approved by the Medical Ethics Committee of Jiaxing Maternal and Child Health Hospital (Approval No: 2023 Research No.041-Quick). Patient data were anonymized. All these procedures were conducted in accordance with the Helsinki Declaration.

### 2.2 Clinical information and definitions

We collected patients’ medical information and outcome data. The medical records included information related to the perinatal period (such as placental abnormalities, fetal distress, and mode of delivery), demographic, clinical characteristics, and feeding approaches [such as gestational age, birth weight, sex, congenital heart disease (CHD), blood transfusion before onset, exposure to antibiotics before onset, anemia, jaundice, assisted ventilation before onset, and type of nutrition], and NEC-related features (such as symptoms at onset, C-reactive protein level at onset, plasma sodium level at onset, dynamic changes in radiographic scores, and antibiotic treatment approach after onset), totaling 18 variables.

Partial definitions of the features and outcome indicators are as follows: (1) Neonatal hyponatremia is defined as plasma sodium (P-Na) <135 mmol/L (22). (2) Dynamic x-ray scoring is based on the Duke abdominal x-ray score (DAAS) for semi-quantitative analysis (7). DAAS scoring method: 0 points, normal intestinal cavity inflation; (a) point, mildly dilated intestinal cavity; (b) points, moderately dilated intestinal cavity or normal inflation with stool-like penetrating shadows; (c) points, intestinal cavity inflation with local and moderate expansion of the intestinal loop; (d) points, intestinal loop separation or local thickening of the intestinal wall; (e) points, multiple separated bowel loops; (f) points, intestinal wall gas accumulation with suspected abnormal abdominal clinical signs; (g) points, fixed or persistent dilatation of bowel loops; (h) points, clinically diagnosed or presence of intestinal wall gas; (i) points, presence of portal vein gas; (j) points, presence of pneumoperitoneum. A transition from a low to a high score within a 6-h interval between consecutive x-ray examinations was defined as deterioration, while the opposite or no change was defined as improvement. The retrospective assessment of DAAS was carried out independently by two radiologists (H Liu and W Bian with 14 and 17 years of experience in pediatric imaging diagnosis, respectively), who were blinded to the patient outcomes, clinical history, and laboratory findings. The discrepancies were resolved through discussion. (3) Neonatal anemia is defined as hemoglobin (Hb) levels of <12 g/dl in the first week after birth, <11 g/dl in the second week, and <9 g/dl in the third week according to the European Consensus Guidelines on the Management of Respiratory Distress Syndrome (23). Throughout the follow-up period, any occurrence of Hb levels meeting these criteria is classified as anemia. (4) Symptoms at onset: Vomiting was defined as an upper gastrointestinal symptom, and bloody stools was defined as a lower gastrointestinal symptom. (5) Indications for transfusion: After a diagnosis of anemia, transfusion therapy was administered based on the clinical judgment of the physician when clinical symptoms such as insufficient weight gain, tachycardia, tachypnea, apnea, and increased oxygen demand were present. (6) Adverse outcomes of NEC are defined as intestinal perforation, the need for surgical intervention due to failure of conservative medical treatment, and inclusion of infants who require surgery but are too critically ill to undergo the procedure. Benign outcomes of NEC are defined as improvement with conservative medical treatment. The observation period extended until the occurrence of the aforementioned outcome.

### 2.3 Statistical analysis

The statistical analysis was conducted using SPSS software (Version 27.0) and R software (Version 4.2.1). Normally distributed continuous data were presented as mean ± standard deviation, while non-normally distributed continuous data were presented as median (M) with lower quartile (QL) and upper quartile (QU). Categorical data were reported as rates or percentages. Statistical significance was set at a *p*-value of <0.05. Chi-square test and Fisher's exact test were used to compare the baseline characteristics between the training set and temporal validation set. The median survival time is calculated using the Kaplan-Meier method, and the Kaplan Meier curves of training set and validation set were plotted. Based on the data from the training set, the least absolute shrinkage and selection operator (LASSO) regression was employed to select the most informative variables. Ten-fold cross-validation was utilized to determine the optimal value of the penalty parameter λ. Subsequently, a multivariate Cox regression model was constructed using the selected variables to predict the adverse outcome of neonates with NEC. The model's nomogram was also plotted. Model evaluation was performed in both the training set and temporal validation set. The Harrell's concordance index (C-index) and time-dependent receiver operating characteristics (tROC) curves were used to evaluate the models’ discrimination ability. We selected several time points for model evaluation using tROC and chose three time points with the best performance.

The consistency between the predicted and actual outcome was graphically assessed with the calibration curve. Decision curve analysis (DCA) was used to assess the clinical usefulness of the model.

## 3 Results

### 3.1 General information

**Figure 1:**
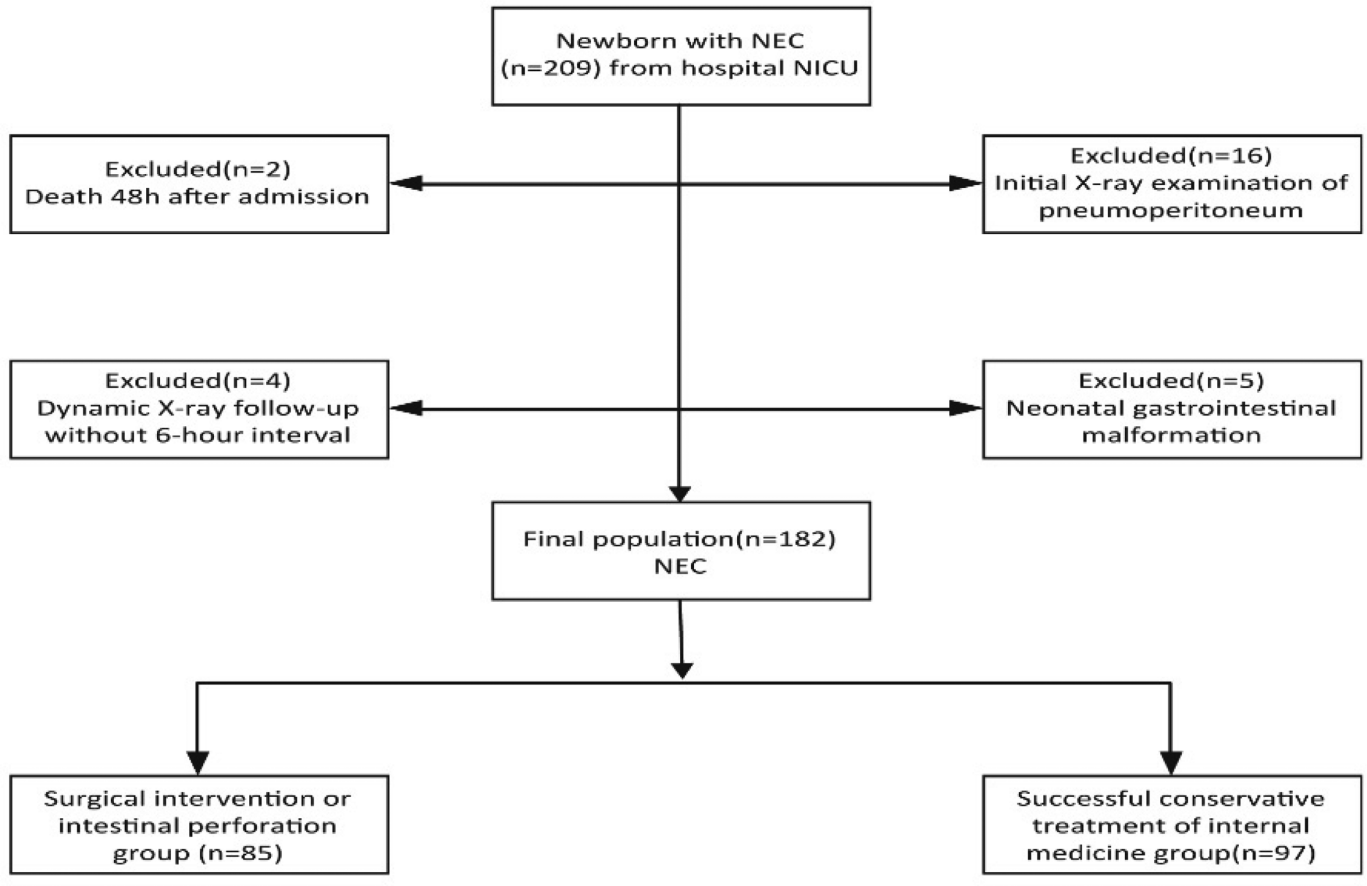
The inclusion-exclusion flowchart of neonates with NEC.

According to the inclusion and exclusion criteria, a total of 182 NEC cases were finally included, resulting in an incidence rate of approximately 0.7% of all NICU cases from January 1, 2017, to December 31, 2022.They were divided into a training set of 148 cases and a validation set of 34 cases based on time series split method. The inclusion-exclusion flowchart is shown in Figure 1.

There was no significant difference in both the selected characteristics between the training group and validation group (Table 1). Some detailed characteristics of NEC neonates are listed in Table 2.

**Table 1:** Baseline characteristics comparison of NEC neonates in training group and validation group.

Note	Variable/assignment	Total (*n* = 182)	Train (*n* = 148)	Valid (*n* = 34)	Statistic	*P*
F1, *n* (%)	Placenta				*χ*² = 0.009	0.92
	0 (normal)	47 (25.82)	38 (25.68)	9 (26.47)		
	1 (anomaly)	135 (74.18)	110 (74.32)	25 (73.53)		
F2, *n* (%)	Fetal distress				*χ*² = 0.067	0.79
	0 (no)	136 (74.73)	110 (74.32)	26 (76.47)		
	1 (yes)	46 (25.27)	38 (25.68)	8 (23.53)		
F3, *n* (%)	Gestational age				*χ*² = 0.005	0.94
	0 (≥37W)	74 (40.66)	60 (40.54)	14 (41.18)		
	1 (<37W)	108 (59.34)	88 (59.46)	20 (58.82)		
F4, *n* (%)	Sex				*χ*² = 0.081	0.77
	0 (female)	87 (47.8)	70 (47.30)	17 (50.00)		
	1 (male)	95 (52.2)	78 (52.70)	17 (50.00)		
F5, *n* (%)	Mode of delivery				*χ*² = 0.666	0.41
	0 (spontaneous labor)	101 (55.49)	80 (54.05)	21 (61.76)		
	1 (cesarean section)	81 (44.51)	68 (45.95)	13 (38.24)		
F6, M (Q₁, Q₃)	Birth weight (g)	2,455.00 (2,170.00, 3,200.00)	2,455.00 (2,207.50, 3,162.50)	2,580.00 (2,115.00, 3,267.50)	*Z* = 0.069	0.94
F7, *n* (%)	Feeding approaches				*χ*² = 0.067	0.79
	0 (formula feeding)	136 (74.73)	110 (74.32)	26 (76.47)		
	1 (mixed feeding)	46 (25.27)	38 (25.68)	8 (23.53)		
F8, *n* (%)	CHD				*χ*² = 0.318	0.57
	0 (no)	72 (39.56)	60 (40.54)	12 (35.29)		
	1 (yes)	110 (60.44)	88 (59.46)	22 (64.71)		
F9, *n* (%)	Blood transfusion				*χ* ² = 1.534	0.21
	0 (no)	87 (47.8)	74 (50.00)	13 (38.24)		
	1 (yes)	95 (52.2)	74 (50.00)	21 (61.76)		
F10, *n* (%)	Antibiotic exposure				*χ*² = 0.000	1.00
	0 (no)	91 (50)	74 (50.00)	17 (50.00)		
	1 (yes)	91 (50)	74 (50.00)	17 (50.00)		
F11, *n* (%)	Anemia				*χ*² = 0.740	0.39
	0 (no)	103 (56.59)	86 (58.11)	17 (50.00)		
	1 (yes)	79 (43.41)	62 (41.89)	17 (50.00)		
F12, *n* (%)	Assisted ventilation				*χ*² = 0.000	1.00
	0 (no)	165 (90.66)	134 (90.54)	31 (91.18)		
	1 (yes)	17 (9.34)	14 (9.46)	3 (8.82)		
F13, *n* (%)	Jaundice				*χ*² = 0.627	0.43
	0 (no)	133 (73.08)	110 (74.32)	23 (67.65)		
	1 (yes)	49 (26.92)	38 (25.68)	11 (32.35)		
F14, *n* (%)	Symptoms at onset				*χ*² = 2.399	0.12
	0 (upper)	96 (52.75)	74 (50.00)	22 (64.71)		
	1 (lower)	86 (47.25)	74 (50.00)	12 (35.29)		
F15, M (Q₁, Q₃)	CRP (mg/L)	76.00 (37.00–119.20)	77.45 (35.12–124.35)	63.65 (41.38–101.30)	*Z* = 0.455	0.65
F16, *n* (%)	P-Na(mmol/L)				*χ*² = 0.383	0.53
	0 (≥135)	93 (51.1)	74 (50.00)	19 (55.88)		
	1 (<135)	89 (48.9)	74 (50.00)	15 (44.12)		
F17, *n* (%)	x-ray scores				*χ*² = 0.096	0.76
	0 (improved/no change)	90 (49.45)	74 (50.00)	16 (47.06)		
	1 (progress)	92 (50.55)	74 (50.00)	18 (52.94)		
F18, *n* (%)	Antibiotic approach				*χ*² = 0.816	0.37
	0 (one)	93 (51.1)	78 (52.70)	15 (44.12)		
	1 (two)	89 (48.9)	70 (47.30)	19 (55.88)		
Time, M (Q₁, Q₃)	Time (day)	10.50 (7.00–16.00)	9.50 (7.00–16.25)	11.75 (7.00–16.25)	*Z* = 0.279	0.78

CRP, c-reactive protein; CHD, congenital heart disease; x-ray scores, dynamic changes in radiographic scores; upper, upper gastrointestinal symptom; lower, lower gastrointestinal symptom; antibiotic: one, metronidazole; two, metronidazole unite vancomycin; time, the median survival time. Feeding approach: formula feeding and mixed feeding.

**Table 2:** Additional characteristics of NEC neonates.

Variables	Total (*n* = 182)			Train (*n* = 148)			Valid (*n* = 34)		
	Statistic			Statistic			Statistic		
	Median	Mean	Range	Median	Mean	Range	Median	Mean	Range
Gestational Age (Day)	250	244.71	98	247	244.49	98	251.5	245.68	94
Birthweight (g)	2,510	2,676.16	2,580	2,455	2,661.14	2,490	2,720	2,741.59	2,260
Day of NEC diagnosis (day)	4	4.23	11	4	4.22	11	4	2.24	7
	*n* (%)			*n* (%)			*n* (%)		
Mortality	4 (2.2%)			4 (2.7%)			0 (0%)		
surgical intervention cases	85 (46.7%)			70 (47.3%)			15 (44.1%)		

*n*, number.

**Figure 2:**
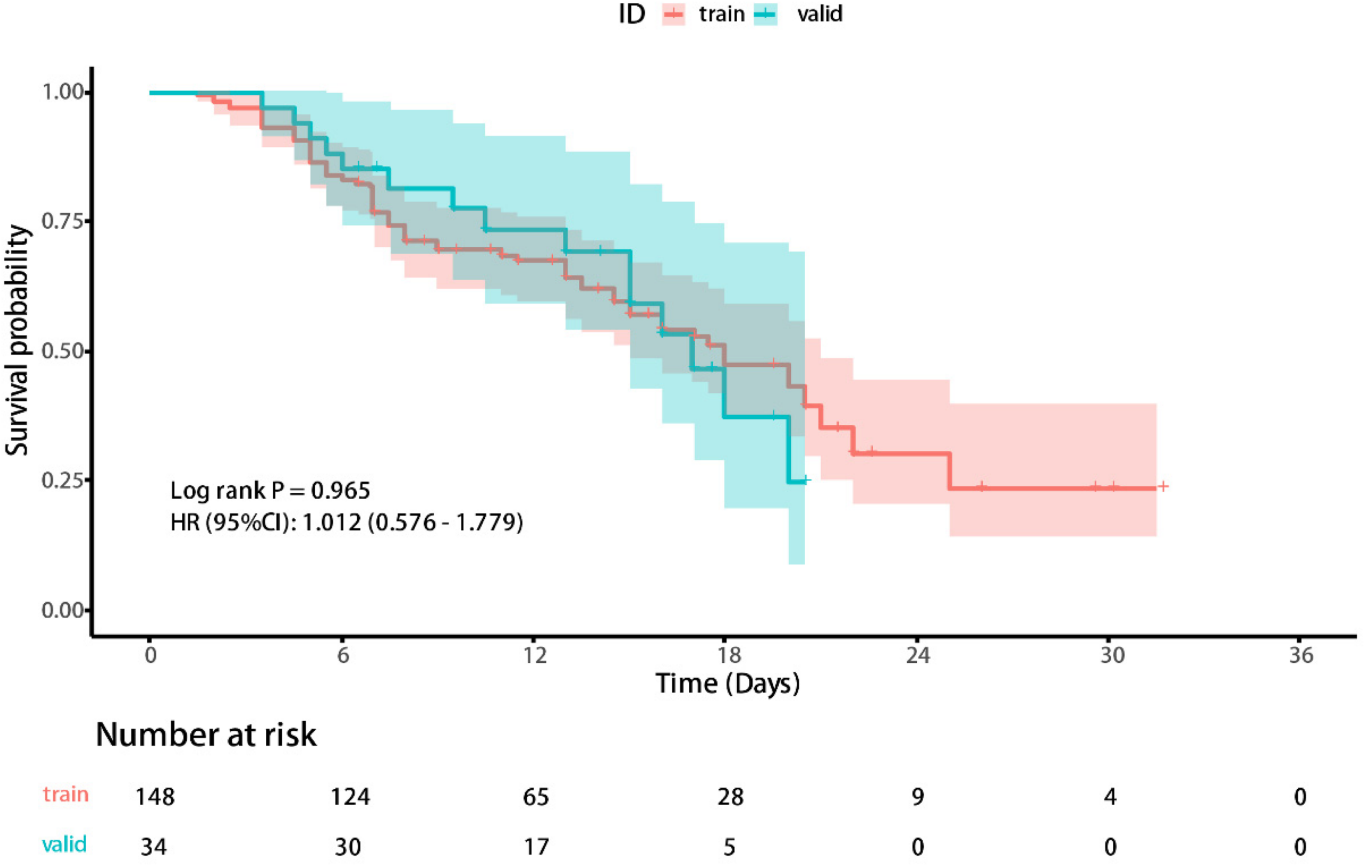
The Kaplan-Meier curves of the training and validation sets.

Ultimately, 46.7% of cases experienced adverse outcomes, with the training set and the validation set having an adverse outcome proportion of 47.3% and 44.1%, respectively. According to Kaplan-Meier method, the median survival time for the included cases was 10.5 (7.00, 16.25) days, with the training set having a median survival time of 9.5 (7.00, 16.75) days and the validation set having a median survival time of 11.75 (7.00, 16.25) days. The Kaplan-Meier curves of the training and validation sets can be seen in Figure 2.

### 3.2 Factor selection and model building

**Figure 3:**
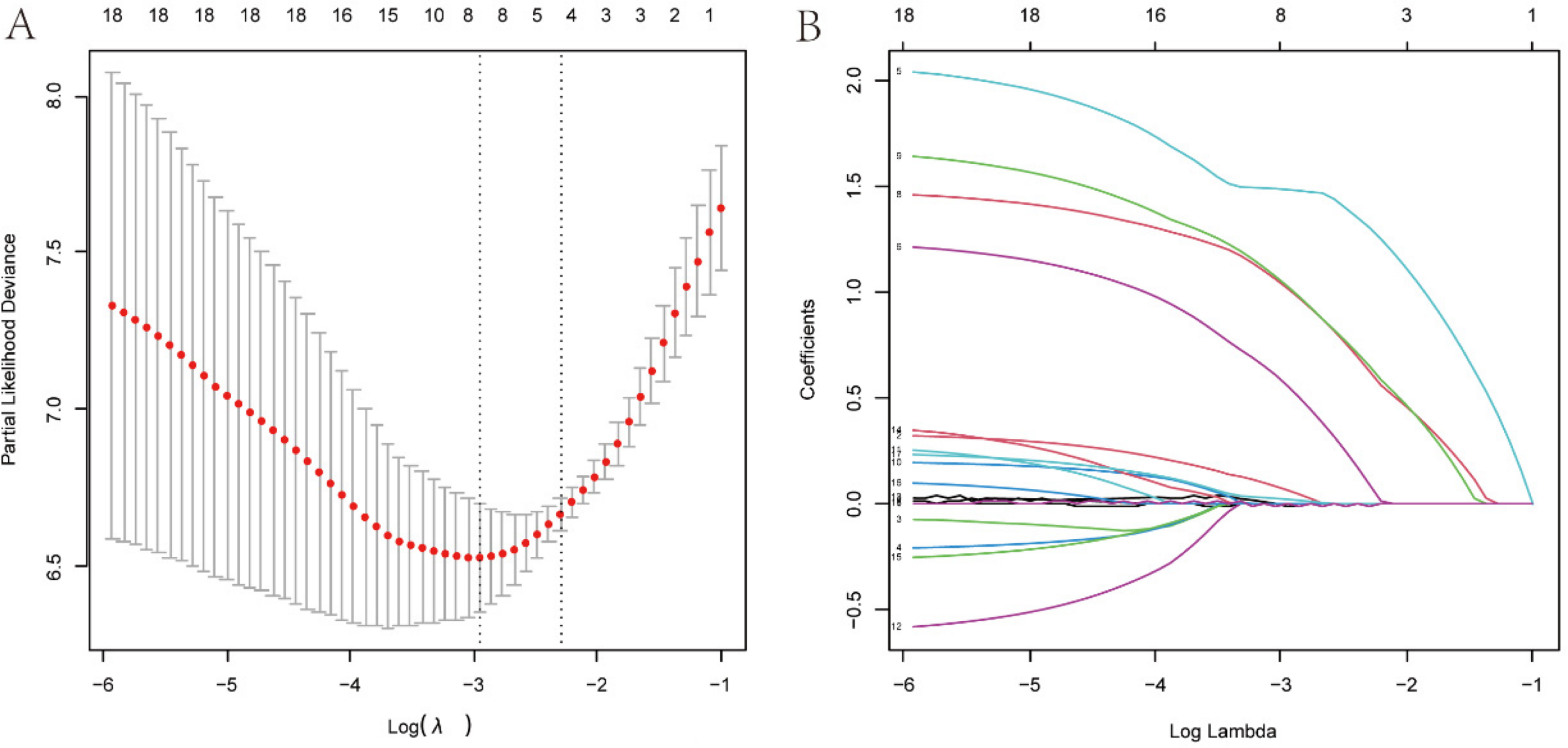
Variables screening based on lasso regression. **(A)** The selection process of the optimum value of the parameter l in the Lasso regression model by cross-validation method. **(B)** The variation characteristics of the coefficient of variables.

The least absolute shrinkage and election operator (LASSO) regression with 10-fold cross validation was used to select the features variables from the included 18 factors, and with the change of penalty coefficient λ (Figure 3A), the coefficients of initially included influencing factors gradually compressed to zero (Figure 3B), achieving the optimal selection of influencing factors.

To find the optimal penalty coefficient λ, the λ value corresponding to the minimum cross-validation error plus one standard error (λmin + 1) was selected as the optimal value for the model. Finally, 8 factors were selected, including weight, CHD, pre-onset blood transfusion, pre-onset antibiotic exposure, CRP at onset, plasma sodium at onset, dynamic x-ray score change, and antibiotic treatment method.

**Figure 4:**
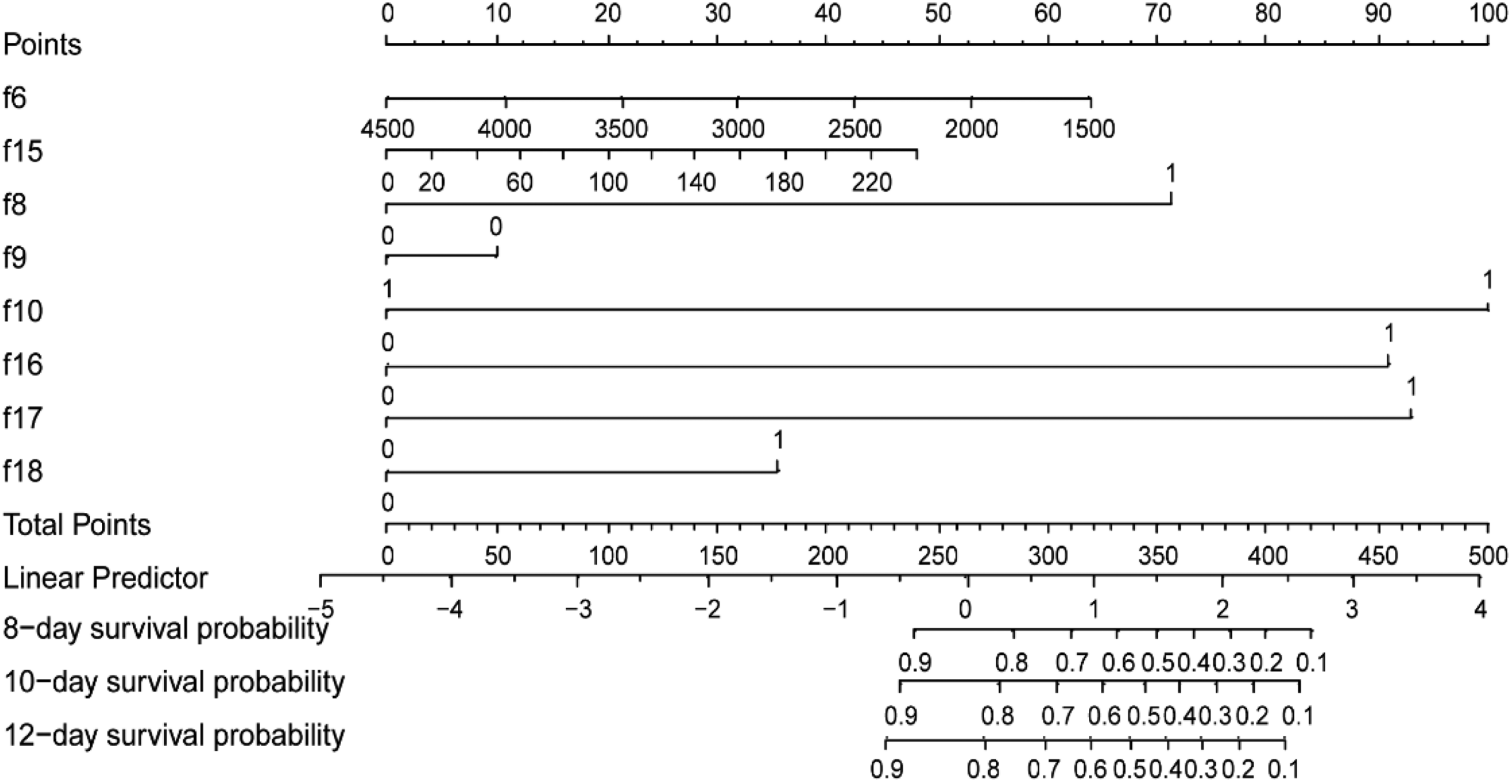
Training set-based nomogram for prediction of time-related adverse outcomes in neonates with NEC. For each patient, sum the scores for the 8 variables to obtain the total score. Then, draw a vertical line from the total points down to the probability scale to determine the predicted probability of adverse NEC outcomes at the three time points. f6, weight (g); f8, congenital heart disease (no = 0, yes = 1); f9, blood transfusion before onset (no = 0, yes = 1); f10, exposure to antibiotics before onset, (no = 0, yes = 1); f15, C-reactive protein level at onset(mg/L) f16, plasma sodium level at onset (mmol/L); f17, dynamic changes in radiographic scores (improved/no change = 0, progress = 1); f18, antibiotic treatment approach after onset (metronidazole = 0; metronidazole unite vancomycin = 1).

Then, the multivariable Cox regression analysis showed that CHD (HR = 3.13, 95%CI:1.75–5.61), antibiotic exposure before onset (HR = 5.52, 95%CI:1.81–16.83), decreased p-Na at onset (HR = 4.73,95%CI:2.61–8.59), and progressive dynamic x-ray scores (HR = 4.90, 95%CI:2.69–8.93) were independent risk factors for adverse outcomes in NEC patients, with statistically significant differences (*P* < 0.05). On the other hand, weight gain (HR = 0.99, 95%CI: 0.98–1.00), blood transfusion before onset (HR = 0.85, 95% CI: 0.29–2.45), and increased CRP before onset (HR = 1.01, 95%CI: 1.00–1.02) were protective factors for NEC outcomes. Combination antibiotic therapy after onset (HR = 1.83, 95%CI: 0.65–5.15) was a risk factor for adverse outcomes in NEC patients. However, these four features did not show statistically significant differences (*P* > 0.05). The detailed results of the multivariate Cox regression analysis of the factors are shown in Table 3. In addition, the factors selected from LASSO regression were used to established a nomogram of Cox regression was developed (Figure 4).

**Table 3:** Multivariate Cox proportional hazards regression of the risk factors for the adverse outcomes of neonates with NEC in the training set.

Variables	Beta	S.E	Z	*P*	HR (95%CI)
Birth Weight	−0.0004	0.0005	−0.72	0.471	0.99 (0.98–1.00)
CRP	0.003	0.002	1.77	0.757	1.01 (1.00–1.02)
CHD					
0					Ref
1	1.22	0.30	4.04	<.001	3.13 (1.75–5.61)
Blood transfusion					
0					Ref
1	−0.17	0.54	−0.31	0.757	0.85 (0.29–2.45)
Antibiotic exposure					
0					Ref
1	1.71	0.57	3.00	0.003	5.52 (1.81–16.83)
P-Na					
0					Ref
1	1.55	0.30	5.11	<.001	4.73 (2.61–8.59)
x-ray scores					
0					Ref
1	1.59	0.31	5.19	<.001	4.90 (2.69–8.93)
Antibiotic therapy					
0					Ref
1	0.61	0.53	1.15	0.250	1.83 (0.65–5.15)

### 3.3 Model evaluation and validation

**Figure 5:**
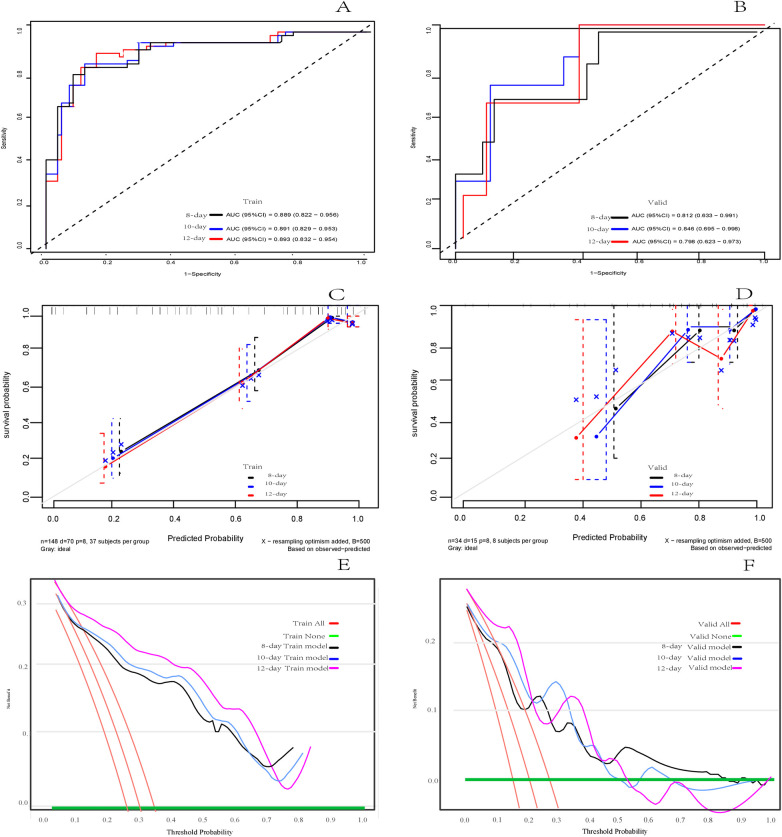
Assessment of the risk prediction model for adverse outcomes in in neonates with NEC. **(A)** The time dependent receiver operating characteristic (ROC) curve of the model in the training set at 8 days, 10 days and 12 days. **(B)** The time dependent ROC curve of the model in the validation set at 8 days, 10 days and 12 days. **(C)** Calibration curve of the model in training set. **(D)** Calibration curve of the model in the validation set. (**E**) Decision curve analysis (DCA) of the model in training set. **(F)** DCA of the model in validation set.

In the training set, the C-index of the model predicting adverse outcomes of NEC was 0.850 (95% CI: 0.804–0.897). The AUCs of tROC at 8 days, 10 days, and 12 days were 0.889 (95% CI: 0.822–0.956), 0.891 (95% CI: 0.829–0.953), and 0.893 (95% CI0.832–0.954), respectively. In the validation set, The C-index of the model was 0.788 (95% CI: 0.656–0.921). The tROC curve showed AUCs at 8, 10, and 12 days were 0.812 (95% CI: 0.633–0.991), 0.846 (95% CI: 0.695–0.998), and 0.798 (95%CI: 0.623–0.973), respectively (Figures 5A,B). The calibration curves of the training set and validation set indicated a fairly good consistency between the actual observed results and those predicted by the model (Figures 5C,D). The DCA curves showed that the net benefit of the model was satisfactory and had a wide range of threshold probabilities of clinical application in training set, and in validation set if the threshold probability less than 45%, using this model to predict the adverse outcomes of NEC can have net benefit (Figures 5E,F).

## 4 Discussion

Early and accurate prediction of the risk of adverse outcomes in NEC patients can provide timely support for clinical decision-making, accurately determine the timing of surgical intervention, and ultimately improve the survival rate. In recent years, a few studies have focused on constructing scoring systems or models for determining risk factors, predicting the risk of adverse outcomes, and making decisions about the timing of surgical intervention in necrotizing enterocolitis. Hassani et al. investigated the predictive factors for surgical treatment of NEC, but their study had a small sample size and lacked validation (5). Qi et al. (24) combined radiological and clinical features to predict the timing of surgical treatment for NEC using the Joint Non-Negative Matrix Factorization method. However, this model did not consider factors such as perinatal, pre-onset, and post-onset treatment measures, making it insufficient for a comprehensive evaluation of NEC outcomes. McElroy et al. constructed a model to predict NEC outcomes using machine learning and artificial intelligence, but their study also faced limitations, including a small sample size. Additionally, some machine learning models may have differences in standard practice, which affects the generalizability of the models, and the interpretability of some models may be challenging (25). Alene et al. (26) investigated the independent predictors of NEC based on Cox regression analysis, but they did not build a complete prediction model yet. Therefore, there is currently no ideal scoring system or model available for application in this field.

We included all preterm and full-term neonates in the study population, which may have contributed to the high risk of atypical NEC. Preterm birth accounted for a large proportion of 108 of the 182 included samples. To ensure comprehensive inclusion of factors, this study incorporated 18 relevant features, including gestational period, demographics, clinical factors, laboratory test results, changes in abdominal x-ray scores, and treatment measures. LASSO regression was used to select features and prevent overfitting, resulting in the selection of 8 features for multivariate COX regression and model building. Since our study aims to evaluate the model's predictive performance for adverse outcomes at different time points, time-based survival analysis maybe the suitable modeling approach.

The multivariable Cox regression analysis revealed that NEC patients with CHD were at a higher risk of adverse outcomes, which aligns with previous studies (27–29). This association may be attributed to the potential impact of systemic hypoxia and abnormalities in systemic circulation, leading to inadequate mesenteric blood flow, which plays a role in the occurrence and progression of NEC. The incidence of CHD in our study population was 60.44%, we also found that full-term neonates with CHD were more likely to develop NEC. Antibiotic exposure before onset was found to be associated with higher likelihood of adverse outcomes, which is consistent with the findings of Zhu et al. (30), This association is likely due to the interference of antibiotic treatment with the establishment of a normal intestinal microbiota in newborns, exacerbating intestinal mucosal barrier damage and contributing to the occurrence and progression of NEC (31). Similarly, although there were no significant differences in the statistical results, there may still be a potential risk of promoting NEC progression when using two antibiotics simultaneously during the treatment of NEC. Therefore, caution should be exercised when using antibiotics in such cases.

In addition, the study found that weight gain had a minimal protective effect and no significant difference was observed, which is not consistent with recent findings (5, 32). This result may be attributed to the limited range of weight distribution in the included infants, as extremely low birth weight infants (birth weight <1,500 g) were not included. Similarly, although preterm blood transfusion may act as a protective factor for the outcome of NEC to some extent, no statistical difference was observed. Yu et al. (7) suggested that transfusion of red blood cells has a protective effect on NEC, while some other studies (33, 34) reached the opposite conclusion. Therefore, larger sample, multicenter prospective cohort studies are needed.

For laboratory findings, we observed a statistically significant increase in the risk of adverse outcomes in NEC patients with hyponatremia. Palleri et al. (22) found that in infants without pneumoperitoneum, each 1 mmol/L decrease in P-Na was associated with a nearly 20% higher likelihood of developing severe NEC. It is speculated that hyponatremia primarily exacerbates intestinal wall damage by activating arginine-vasopressin and antidiuretic hormone, leading to water retention. This finding is consistent with our results. A previous study has demonstrated an association between severe NEC cases and increased CRP levels (35). However, in our study, the impact of CRP was relatively limited and not statistically significant. This may be due to the fact that CRP reflects the overall inflammatory status of the body, and the cases in our study may have had other clinical factors contributing to increased CRP levels besides NEC.

Abdominal x-ray examination is an effective auxiliary tool in the diagnosis and treatment evaluation of necrotizing enterocolitis (NEC). In this study, the DAAS scoring system, which indicates disease progression, was found to be an independent risk factor for adverse outcomes in NEC patients. Common abdominal x-ray findings in NEC include intestinal distension, thickened intestinal walls, bowel loop separation, pneumatosis intestinalis, and even pneumoperitoneum. The DAAS scoring system assigns scores ranging from 0 to 10 based on these radiographic findings. Yu et al. (7) reported that a DAAS score ≥7 combined with laboratory indicators, had a sensitivity of 12.8% and specificity of 100% for diagnosing adverse outcomes in NEC. Although the diagnostic performance is not high, we found that observing its dynamic changes can sensitively detect subtle alterations in the course of the disease, providing prognostic information for NEC patients.

Although the multivariable Cox regression analysis suggested that some features had a small effect on the adverse outcomes of NEC and that some differences were not statistically significant, this study still included all eight variables selected by LASSO regression to construct the nomogram based on Cox regression (36–38). This decision was based on clinical practice and previous reports that focused on NEC risk factors. The C-index and the AUCs of tROC demonstrated good discrimination ability and predictive performance in both the training set and validation set. Particularly, the AUCs of tROC at the 10-day time point showed the best performance, indicating that the model effectively predicts the occurrence of adverse outcomes at this time point. Kaplan-Meier analysis showed the median survival time for the included cases was 10.5 (7.00, 16.25) days, Therefore, selecting a time point before the median survival time that can effectively predict adverse outcomes will support early clinical decision-making. The ROC's AUC at the 8-day time point demonstrated acceptable predictive performance, suggesting early identification of adverse outcomes risk in NEC patients compared to the 10-day time point, making it possible to early identify adverse outcomes of NEC, thereby further supporting timely clinical decision-making and determination of the optimal timing for surgical intervention.

However, our study does have several limitations. Firstly, being a retrospective study, it may introduce selection bias and unreported confounding factors. Secondly, the overall sample size of the study is relatively small, while we investigated many variables. Although LASSO regression was used for variable selection, there may still be a risk of bias. Thirdly, this study was conducted at a single center, which may introduce overall bias in the assessment and treatment of NEC. The validation set comes from the same center, lacking independent external validation. Fourthly, due to the rapid progression of NEC, some variables in this study were only assessed at a few time points. This may underestimate or overlook the impact of changes in certain risk factors on the overall prognosis. Although the COX model demonstrated relatively high predictive performance at the 8-day point, issues such as delayed prediction of adverse outcomes and potential implementation barriers in clinical practice remain. Therefore, future research should include large-sample, multicenter cohort studies for further investigation and validation.

## 5 Conclusion

This study developed a LASSO-Cox proportional hazards regression-based nomogram to predict adverse outcomes in NEC. The model exhibited good predictive performance, offering a simple, accessible, and cost-effective tool for early and effective identification of adverse outcomes, facilitating timely surgical intervention, and improving patient prognosis.

## Data Availability

The original contributions presented in the study are included in the article/Supplementary Material, further inquiries can be directed to the corresponding author.
